# Clinical phenotypes of children undergoing adenotonsillectomy: a cluster analysis based on comorbidities and Its association with surgical strategy and length of stay

**DOI:** 10.3389/fsurg.2026.1875200

**Published:** 2026-08-17

**Authors:** Chen Liu, Jiali Zhang, Liu Yang, Lifang Tan

**Affiliations:** 1 Department of Otorhinolaryngology, Maternal and Child Health Hospital of Hubei Province, Tongji Medical College, Wuhan, China; 2 Department of Otolaryngology Head and Neck Surgery, The 910th Hospital of the Chinese People’s Liberation Army Joint Logistic Support Force, Quanzhou, Fujian, China; 3 Zhuhai Hospital of Integrated Traditional Chinese and Western Medicine, Zhuhai, China

**Keywords:** adenotonsillectomy, cluster analysis, length of stay, pediatric obstructive sleep apnea, phenotype

## Abstract

**Objective:**

To identify clinical phenotypes among children undergoing surgery for adenotonsillar hypertrophy using unsupervised cluster analysis based on comorbidities, and to explore the association of phenotypes with obstructive sleep apnea (OSA) severity, surgical procedures, and length of hospital stay (LOS).

**Methods:**

This retrospective cohort study included 199 consecutive children who underwent adenoidectomy or adenotonsillectomy between August 2023 and April 2026. Nine binary comorbidity variables were extracted. K-modes clustering was used to partition patients into phenotypic groups. Demographic characteristics, OSA severity, surgical procedures, and LOS were compared. Multivariable negative binomial regression identified independent predictors of LOS.

**Results:**

Four phenotypes emerged: allergic-inflammatory (*n* = 48), sinusitis-dominant (*n* = 90), otitis-prone (*n* = 22), and hypertrophy-only (*n* = 39). Age, OSA prevalence, and severity varied significantly among clusters (*P* < 0.05). Tympanostomy tube insertion was concentrated in the otitis-prone cluster (86.4%, *P* < 0.001). LOS differed modestly (*P* = 0.043), with the otitis-prone cluster staying longest. Only surgical extent independently predicted LOS: standard T&A (IRR 1.27, 95% CI 1.07–1.51) and extended surgery (IRR 1.45, 95% CI 1.01–2.03).

**Conclusions:**

Comorbidity-based clustering reveals four clinically meaningful phenotypes differing in age, OSA, and otologic surgery needs. LOS is driven by surgical complexity, yet phenotyping aids preoperative planning.

## 1. Introduction

Adenotonsillectomy (T&A) is the first-line surgical treatment for pediatric obstructive sleep apnea (OSA) and symptomatic adenotonsillar hypertrophy (1, 2). The prevalence of OSA in children is estimated at 1%–5%, with adenotonsillar hypertrophy being the most common etiology (3, 4). Children undergoing T&A frequently present with a constellation of upper airway comorbidities, including chronic rhinosinusitis, allergic rhinitis, otitis media with effusion (OME), and structural nasal abnormalities (5, 6). These comorbidities may not only influence the decision to operate but also affect perioperative outcomes and healthcare utilization (7, 8).

Current clinical guidelines recommend individualized treatment based on OSA severity assessed by polysomnography (PSG) or clinical evaluation (2, 9). Although PSG remains the gold standard, its accessibility varies across settings, and standardized severity documentation may be incomplete even when PSG is performed (10). Furthermore, the heterogeneity of comorbidity profiles among surgical candidates has not been systematically characterized. Most studies have examined single comorbidities in isolation (11, 12), overlooking the fact that many children suffer from multiple overlapping conditions. This is a critical gap because the pattern of comorbidities—rather than any single diagnosis—might better reflect the underlying pathophysiological endotype and predict clinical trajectories (13).

Cluster analysis, an unsupervised machine learning technique, has been successfully applied in respiratory medicine to identify phenotypes with distinct treatment responses and prognoses, such as in asthma and chronic rhinosinusitis (14, 15). In otolaryngology, clustering has been used to classify OSA patients based on polysomnographic parameters (16, 17). To date, no study has applied comorbidity-based clustering to pediatric adenotonsillectomy candidates. Such an approach could uncover hidden clinical phenotypes that inform surgical decision-making, resource allocation, and perioperative risk stratification.

Therefore, we conducted this retrospective cohort study to: (1) identify distinct clinical phenotypes based on routine comorbidity data using unsupervised clustering; (2) compare these phenotypes in terms of demographics, OSA severity, and surgical management; and (3) evaluate whether phenotype independently predicts length of hospital stay (LOS) after adjusting for surgical factors.

## 2 Methods

### 2.1 Study design and participants

This retrospective study included consecutive pediatric patients (≤18 years) who underwent adenoidectomy or adenotonsillectomy at the Department of Otolaryngology, Maternal and Child Health Hospital of Hubei Province, between August 31, 2023 and April 6, 2026.The study involved de-identified, retrospectively collected data. According to the institutional policy of the Maternal and Child Health Hospital of Hubei Province, formal ethics approval and a specific approval number are not required for such retrospective studies. Written informed consent from patients was waived due to the retrospective nature and use of anonymized data.

Patients were identified from the hospital discharge database. Inclusion criteria were: (1) primary surgical procedure of adenoidectomy or adenotonsillectomy (with or without additional procedures); (2) complete medical records. Exclusion criteria: (1) age >18 years; (2) missing key diagnostic or procedural data.

### 2.2 Data collection and variable extraction

The following data were extracted: age (converted to months), sex, discharge diagnoses, surgical procedures, and length of stay (LOS, days). Based on discharge diagnoses, nine binary comorbidity variables were created (present = 1, absent = 0): sinusitis, allergic rhinitis, otitis media (including acute, secretory, and bullous myringitis), turbinate hypertrophy, nasal stenosis, nasal septal deviation, tonsillitis, pharyngeal stenosis/uvulopalatal elongation, and upper respiratory infection/mycoplasma infection.

OSA was defined as the presence of relevant terms in the discharge diagnosis (2). OSA severity was extracted as “mild”, “moderate”, “severe”, or “ungraded” based on explicit documentation.

Surgical procedures were categorized into three ordered categories: (0) isolated adenoidectomy; (1) standard tonsillectomy with adenoidectomy; (2) extended surgery additionally involving uvulopalatoplasty, turbinate reduction, or radiofrequency ablation. Concurrent tympanostomy/ventilation tube insertion was recorded as a separate binary variable.

### 2.3 Clustering analysis

K-modes clustering was performed on the nine binary comorbidity variables using the klaR package in R (18). The optimal number of clusters (k) was determined by examining the elbow plot of total within-cluster distance and the average silhouette width over k = 2 to 8, balancing statistical optimality with clinical interpretability. Clusters were labeled descriptively based on predominant comorbidity patterns.

### 2.4 Statistical analysis

Continuous variables are presented as median (IQR) and compared using the Kruskal–Wallis test. Categorical variables are presented as frequencies (%) and compared using Fisher's exact test (simulated P-value, 10,000 replicates). *post-hoc* pairwise comparisons of LOS were conducted with Wilcoxon rank-sum test and Bonferroni correction.

Multivariable negative binomial regression was used to identify independent predictors of LOS, with candidate variables including cluster assignment, age, sex, surgical procedure category, and concurrent tympanostomy tube insertion. Stepwise selection based on AIC was used. Incidence rate ratios (IRR) with 95% CI are reported. All analyses were performed in R 4.3.1. A two-sided *P* < 0.05 was considered significant.

## 3 Results

### 3.1 Study population

A total of 199 eligible children were included. Median age was 74 months (IQR 48–99), and 124 (62.3%) were male. OSA was diagnosed in 60.8% (*n* = 121), with mild, moderate, and severe OSA accounting for 24.6%, 8.0%, and 3.5% respectively; 24.6% were ungraded. Most children underwent standard T&A (77.9%), followed by isolated adenoidectomy (18.6%) and extended surgery (3.5%). Concurrent tympanostomy tube insertion was performed in 12.6%. These demographics are consistent with typical pediatric T&A populations (19).

### 3.2 Phenotype identification

**Figure 1:**
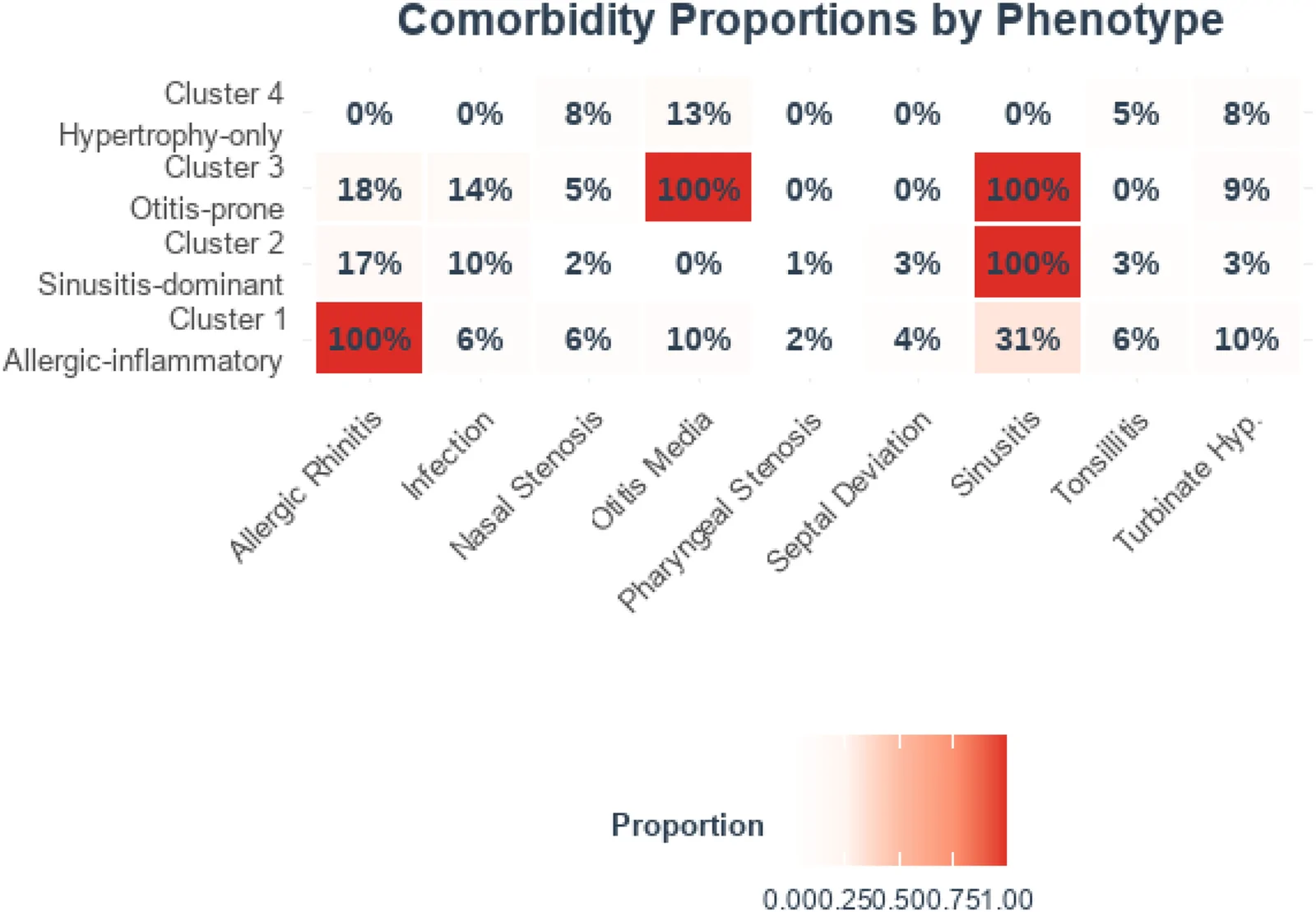
Comorbidity proportion heatmap by cluster. Color gradient represents the proportion (0%–100%) of each comorbidity within the cluster; values are shown as percentages. Cluster 1 (allergic-inflammatory) has high sinusitis and allergic rhinitis; Cluster 2 (sinusitis-dominant) has extremely high sinusitis but low allergy; Cluster 3 (otitis-prone) has nearly universal otitis media and sinusitis; Cluster 4 (hypertrophy-only) shows low proportions overall.

K-modes clustering identified four distinct phenotypes (Figure 1). Cluster 1 (*n* = 48, 24.1%) had high sinusitis (100%), high allergic rhinitis (31.2%), and moderate otitis media (10.4%) — the allergic-inflammatory phenotype. Cluster 2 (*n* = 90, 45.2%) had the highest sinusitis rate (100%) but low allergic rhinitis (3.3%) and otitis media (2.2%) — the sinusitis-dominant phenotype. Cluster 3 (*n* = 22, 11.1%) exhibited 100% otitis media and 100% sinusitis with low allergy (9.1%) — the otitis-prone phenotype. Cluster 4 (*n* = 39, 19.6%) had low comorbidities except for relatively higher structural abnormalities — the hypertrophy-only phenotype. The comorbidity heatmap is shown in Figure 1.

### 3.3 Clinical characteristics by cluster

**Figure 2:**
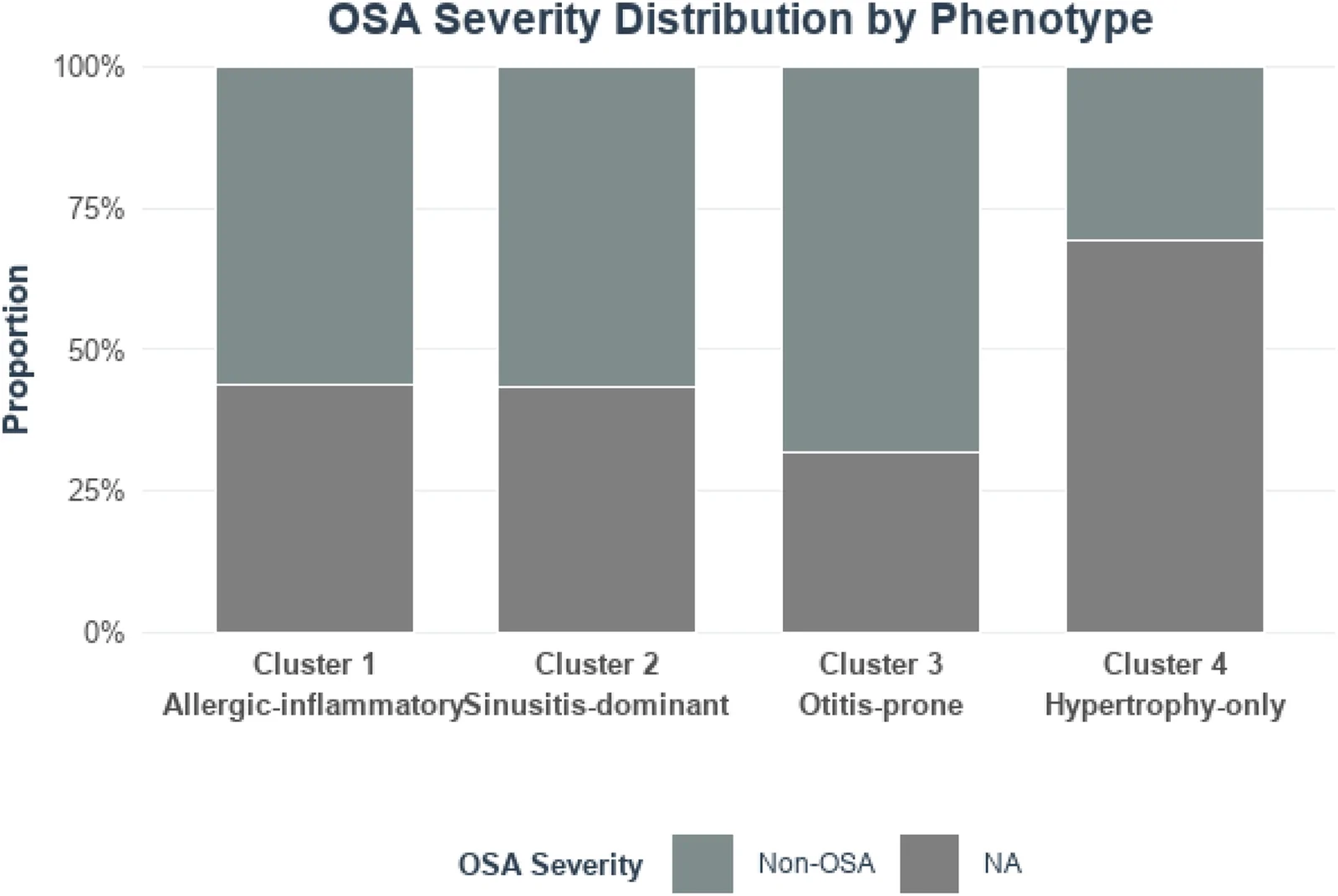
Distribution of OSA severity by cluster (stacked proportion bar plot). Each bar represents a cluster; colors indicate OSA severity categories (Non-OSA, Mild, Moderate, Severe, Ungraded). The distribution differed significantly (Fisher's exact test, *P* = 0.017).

Table 1 summarizes demographics and clinical features. Age differed significantly (*P* = 0.046), with Cluster 3 being the youngest (median 60 months vs. 74.5–84.5). Sex distribution did not differ (*P* = 0.533). OSA prevalence varied significantly (*P* = 0.017), highest in Cluster 4 (69.2%) and lowest in Cluster 3 (31.8%). Severity grades also differed (*P* = 0.017): moderate OSA was most frequent in Cluster 4 (15.4%), and severe OSA was absent in Cluster 1. Figure 2 shows the severity distribution.

**Table 1:** Demographic and Clinical Characteristics by Cluster.

Variable	Cluster 1 (*n* = 48)	Cluster 2 (*n* = 90)	Cluster 3 (*n* = 22)	Cluster 4 (*n* = 39)	*P*-value
Age, months, median (IQR)	84.5 (58.0–111.5)	74.5 (50.0–97.5)	60.0 (45.0–82.0)	78.0 (65.0–109.5)	0.046
Female, n (%)	21 (43.8)	31 (34.4)	6 (27.3)	13 (33.3)	0.533
OSA diagnosis, n (%)	21 (43.8)	39 (43.3)	7 (31.8)	27 (69.2)	0.017
OSA severity, n (%)					0.017
Mild	7 (14.6)	21 (23.3)	2 (9.1)	11 (28.2)	
Moderate	4 (8.3)	1 (1.1)	0 (0.0)	6 (15.4)	
Severe	0 (0.0)	3 (3.3)	1 (4.5)	1 (2.6)	
Ungraded	10 (20.8)	14 (15.6)	4 (18.2)	9 (23.1)	
Surgical procedure, n (%)					0.300
Isolated adenoidectomy	8 (16.7)	15 (16.7)	6 (27.3)	8 (20.5)	
Standard T&A	36 (75.0)	74 (82.2)	16 (72.7)	29 (74.4)	
Extended surgery	4 (8.3)	1 (1.1)	0 (0.0)	2 (5.1)	
Tympanostomy tube, n (%)	3 (6.2)	0 (0.0)	19 (86.4)	3 (7.7)	<0.001
LOS, days, median (IQR)	5 (4–5.25)	5 (4–5)	5 (5–6)	5 (4–5.5)	0.043

Continuous variables: Kruskal–Wallis test; categorical: Fisher's exact test with simulated P.

**Figure 3:**
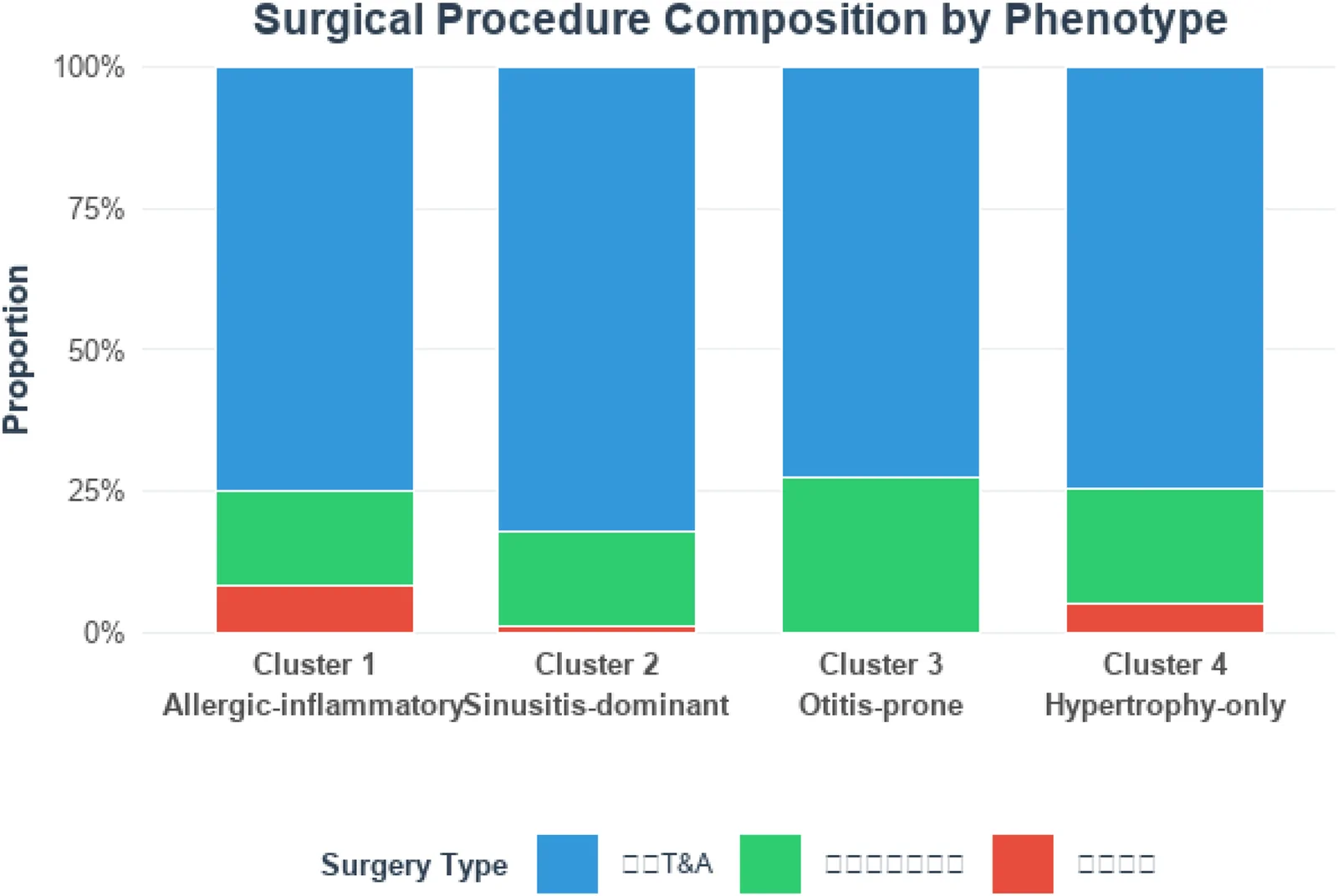
Surgical procedure composition by cluster (stacked proportion bar plot). Isolated adenoidectomy, standard T&A, and extended surgery are shown. No significant difference in procedure distribution was found (*P* = 0.300).

Surgical procedure distribution was similar across clusters (*P* = 0.300). However, tympanostomy tube insertion was overwhelmingly concentrated in Cluster 3 (86.4% vs. 0%–7.7%, *P* < 0.001, Figure 3).

**Figure 4:**
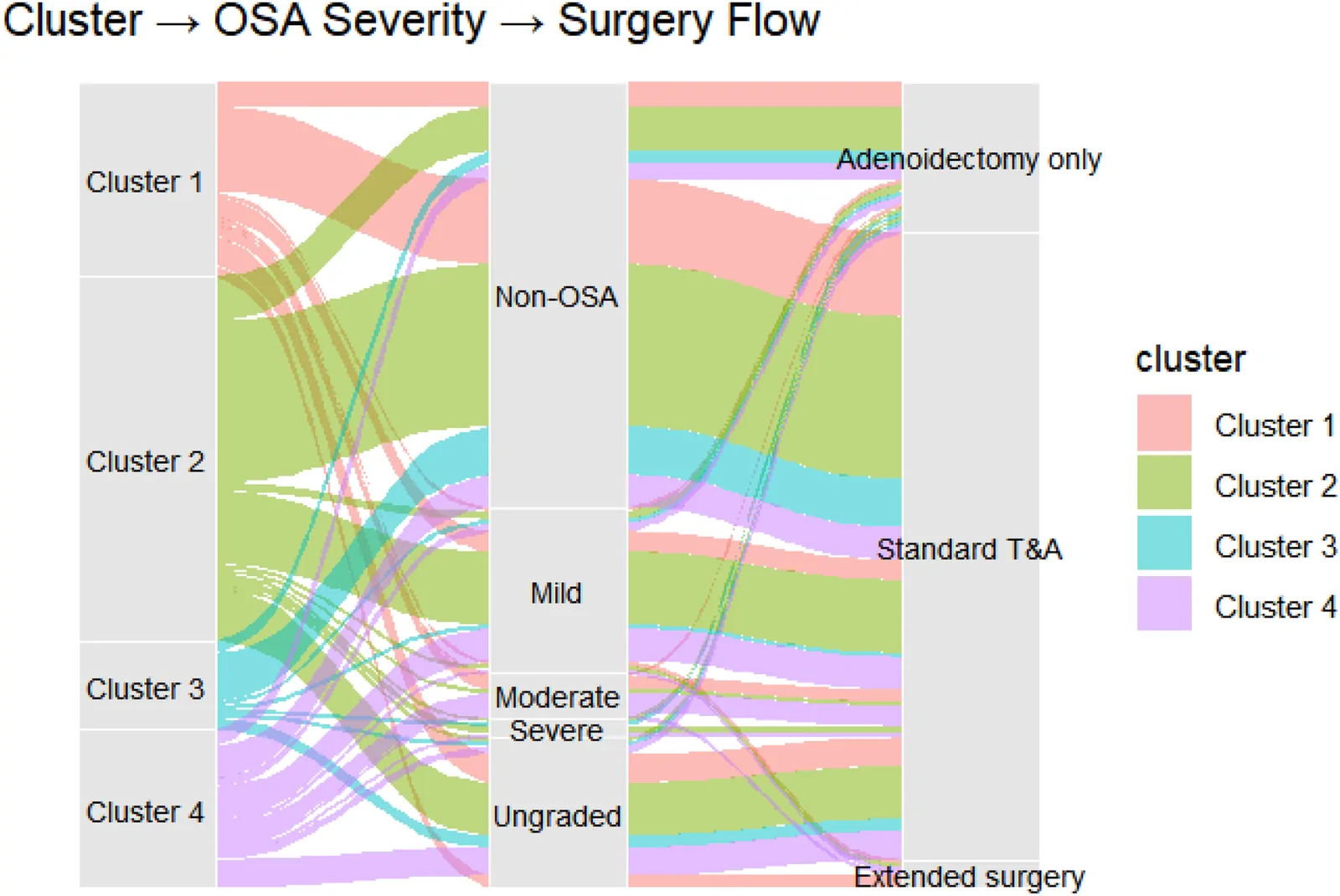
Sankey flow diagram: Cluster → OSA Severity → Surgical Procedure. The width of flows represents the number of patients transitioning from phenotype through OSA severity to surgery type.

The Sankey flow diagram (Figure 4) illustrates the transition from phenotypes to OSA severity grades and subsequently to surgical procedures. The allergic-inflammatory and sinusitis-dominant clusters (Clusters 1 and 2) flowed predominantly toward the “non-OSA” and “ungraded” categories, whereas the hypertrophy-only cluster (Cluster 4) was disproportionately directed toward “OSA,” especially “mild” and “moderate” grades. Regardless of OSA severity, standard T&A remained the dominant surgical procedure across all clusters. Extended surgery, though infrequent, tended to originate from Clusters 1 (allergic-inflammatory) and 4 (hypertrophy-only). Tympanostomy tube insertions were almost exclusively linked to the otitis-prone cluster (Cluster 3) via any OSA severity category.

### 3.4 Length of stay and predictors

**Figure 5:**
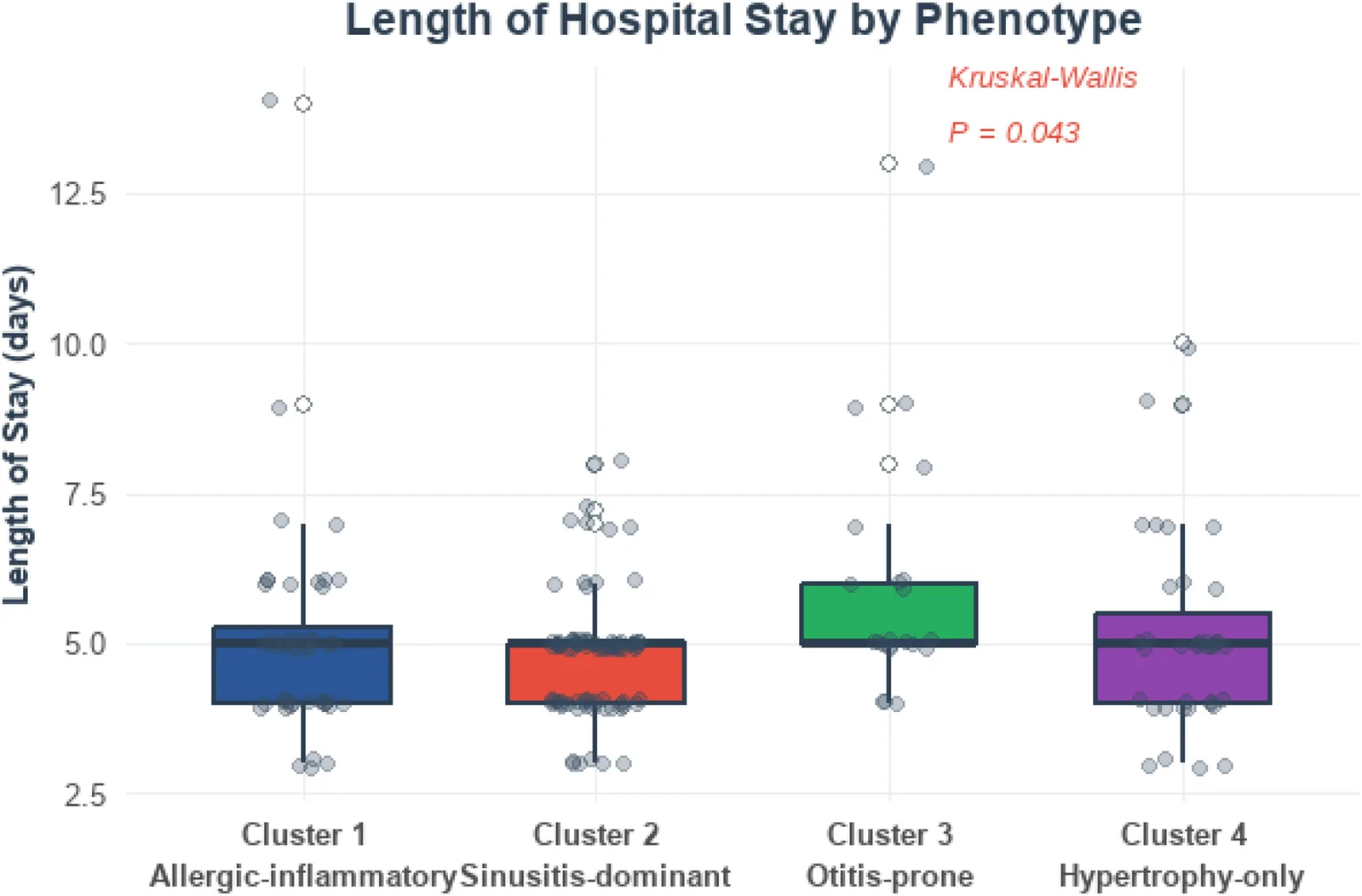
Length of hospital stay by cluster. Box plots overlaid with jittered data points. Kruskal–Wallis test *P* = 0.043. In *post-hoc* analysis, Cluster 3 had significantly longer LOS than Cluster 2 (adjusted *P* = 0.017).

Median LOS was 5 days (IQR 4–5). LOS varied modestly across clusters (*P* = 0.043, Figure 5), with Cluster 3 significantly longer than Cluster 2 (adjusted *P* = 0.017). In multivariable negative binomial regression (Table 2), only surgical extent independently predicted LOS. Compared to isolated adenoidectomy, standard T&A increased LOS by 27% (IRR 1.27, 95% CI 1.07–1.51, *P* = 0.007), and extended surgery by 45% (IRR 1.45, 95% CI 1.01–2.03, *P* = 0.036). Concurrent tympanostomy tube insertion showed borderline significance (IRR 1.20, *P* = 0.050). Cluster assignment, age, and sex were not retained in the final model.

**Table 2:** Multivariable Negative Binomial Regression for Length of Stay.

Variable	IRR	95% CI	*P*-value
Surgery type (ref: isolated adenoidectomy)			
Standard T&A	1.27	1.07–1.51	0.007
Extended surgery	1.45	1.01–2.03	0.036
Tympanostomy tube (yes vs. no)	1.20	1.00–1.43	0.050

Variables not retained: cluster assignment, age, sex.

## 4 Discussion

This study is the first to apply unsupervised clustering to pediatric adenotonsillectomy candidates based solely on comorbidity profiles, revealing four clinically meaningful phenotypes. These phenotypes differed in age, OSA prevalence, and the need for concurrent otologic surgery, yet LOS was primarily driven by surgical complexity rather than phenotype itself.

The allergic-inflammatory phenotype (Cluster 1) likely represents children with atopic predisposition, which is known to exacerbate both rhinosinusitis and OSA (20, 21). The sinusitis-dominant phenotype (Cluster 2) may reflect isolated infectious processes, such as recurrent upper respiratory infections, a common indication for T&A (22). The otitis-prone phenotype (Cluster 3), consisting of the youngest children, aligns with the well-established association among early-life eustachian tube dysfunction, OME, and adenoid hypertrophy (23).The 100% co-occurrence of sinusitis and otitis media in this cluster merits particular discussion. In pediatric populations, OME is rarely an isolated condition; rather, it frequently coexists with sinonasal inflammation due to shared anatomical pathways and the unifying “united airway” concept. The eustachian tube, which connects the nasopharynx to the middle ear, is both a potential conduit for pathogens from inflamed sinuses and a structure whose dysfunction impairs middle ear ventilation and sinonasal drainage alike (24, 25). Adenoid hypertrophy—itself a hallmark indication for surgery—further compounds this relationship by simultaneously obstructing the eustachian tube orifice and the posterior choanae, worsening both OME and sinusitis. The near-universal presence of sinusitis in the otitis-prone cluster should therefore be interpreted not as documentation bias or statistical artifact, but as a clinically expected consequence of shared pathophysiological mechanisms. This observation validates the clustering algorithm, which correctly identified the natural tendency of sinusitis and otitis media to co-aggregate in surgically treated children.The hypertrophy-only phenotype (Cluster 4) with minimal inflammation but the highest OSA prevalence may represent the “pure” mechanical form of adenotonsillar hypertrophy, where surgery is most directly indicated (26).

The lack of significant difference in surgical procedure type across clusters (except tympanostomy tube) suggests that at our institution, the decision to perform T&A or extended surgery relies mainly on objective tonsil/adenoid size and OSA severity rather than the broader comorbidity profile. However, the strong enrichment of tympanostomy tube insertion in the otitis-prone cluster validates the clustering approach and suggests that automated phenotyping could standardize preoperative evaluation—for example, by flagging patients likely to need tympanometry and otologic intervention (27).

Our finding that LOS is independently predicted by surgical extent rather than cluster assignment is consistent with the understanding that more extensive procedures require longer postoperative monitoring (28). The borderline significance of tympanostomy tube placement (*P* = 0.05) indicates that adding an otologic procedure may modestly prolong hospitalization, a factor that should be considered in enhanced recovery protocols.

The study has limitations. First, its single-center, retrospective design limits generalizability. Second, although OSA diagnosis and severity grading were based on polysomnographic findings, the retrospective reliance on discharge diagnoses precluded independent review of raw PSG parameters. Furthermore, 24.6% of OSA cases lacked explicit severity documentation despite PSG confirmation, potentially attenuating or inflating observed severity differences across clusters. Standardized prospective PSG data collection would enhance severity stratification in future studies. Third, we lacked postoperative outcomes such as symptom improvement or complications, precluding assessment of whether phenotype predicts treatment success. Fourth, the small size of Cluster 3 (*n* = 22) reduced statistical power and may limit the generalizability of this phenotype. The 100% co-occurrence of sinusitis and otitis media observed in this cluster, while clinically plausible, warrants prospective validation to exclude potential selection bias inherent to surgical cohorts. Specifically, children with OME who are referred for adenoidectomy may be more likely to receive comprehensive diagnostic workup, inflating the apparent comorbidity burden. Future studies incorporating cluster stability metrics (e.g., bootstrapping or cross-validation) and comparing surgical vs. medically managed cohorts would help confirm whether this cluster is a true phenotype or a context-dependent finding.

Despite these limitations, this study demonstrates that comorbidity-based clustering offers a simple, clinically accessible tool for subclassifying children undergoing adenotonsillectomy. As electronic health records and machine learning become more prevalent, such phenotyping could be automated at the point of care to support personalized surgical planning and resource allocation (23).

## 5 Conclusion

Comorbidity-based cluster analysis identifies four distinct phenotypes among children undergoing adenotonsillar surgery. These phenotypes differ in age, OSA prevalence, and need for concurrent tympanostomy tube insertion. Although LOS is driven by surgical extent, phenotyping may aid in preoperative planning, particularly for the otitis-prone cluster. This approach represents a step toward precision medicine in pediatric otolaryngology.

## Data Availability

The original contributions presented in the study are included in the article/Supplementary Material, further inquiries can be directed to the corresponding authors.
